# Evaluation of the outcomes of equity-deserving individuals receiving services and support from integrated substance use health and mental health services: a pilot study protocol

**DOI:** 10.3389/fpsyt.2024.1425514

**Published:** 2024-12-10

**Authors:** Hanie Edalati, Christina Katan, Sheena Taha

**Affiliations:** Canadian Centre on Substance Use and Addiction (CCSA), Ottawa, ON, Canada

**Keywords:** substance use health, mental health, integrated services and support, equity-deserving populations, equity-oriented care

## Abstract

**Background:**

Given the close relationship that can exist between substance use health and mental health (SUHMH) concerns, the need for more integrated services and support has been identified. However, research on the effective integration of SUHMH services and their impact on outcomes of individuals accessing them remains limited. In particular, the unique outcomes of individuals facing significant structural inequities in the health care system, i.e., Indigenous Peoples in Canada, including First Nations, Inuit and Métis (FNIM), and equity-deserving (ED) groups, have not been evaluated while receiving integrated SUHMH services. This paper describes the protocol for a pilot research project, which will evaluate the change in clinical and social outcomes of individuals receiving integrated SUHMH services in relation to their intersectionality status, a linear score ranging from identifying with no FNIM and ED groups to identifying with one or multiple groups.

**Methods:**

This study recruits 100 individuals who receive SUHMH services and support from a community health center in Ottawa, Canada and assessed their FNIM and ED status and clinical and social outcomes at baseline and three-month follow-up. At the time of writing this manuscript, the baseline data collection was completed. Follow up assessment occurs three months after baseline data was collected. A smaller group of these participants will be purposefully selected match the proportion of FNIM and ED groups from the two-time assessment. They will be invited to participate in a post-data analysis validation consultation session to ensure that the findings are reflective of the experiences of individuals receiving SUHMH services, alternative interpretations are brought forward, and implications are driven by those who will be most impacted. This consultation may also inform knowledge mobilization activities and future studies. This study also recruited staff in different roles from the providing center to rate the implementation of key dimensions of equity-oriented care into their practices and their level and capability to provide integrated services and support to individuals with cooccurring substance use disorders and mental illnesses.

**Discussion:**

The results of this study will inform integrated SUHMH services by emphasizing equity and inclusive approaches, and engagement with the community. Substance use health; Mental health; Integrated services and support; Equity-deserving populations; Equity-oriented care.

## 1 Introduction

Evidence from several studies has indicated that substance use disorders often cooccur with mental illnesses (1–3). Individuals living with cooccurring substance use disorders and mental illnesses experience poorer treatment outcomes, shorter stays in treatment, lower rates of treatment completion, and higher rates of recurrence and rehospitalization after treatment (4–6) than those with substance use disorders only. Cooccurring mental illnesses also create several challenges for people who need both substance use health and mental health (SUHMH) services. Furthermore, individuals living with cooccurring substance use disorders and mental illnesses may have difficulty identifying and accessing available services, coordinating and navigating them, and receiving appropriate SUHMH services (7, 8). Given these concerns and the close relationship that can exist between substance use health and mental health, the need for more integrated services and support has been identified (9).

Integrated SUHMH care is generally defined as *“the degree of linkage or collaboration between substance use and mental health—ranging from cooperation to amalgamation—at both the service and system levels”* (10). There are many types and levels of integration (11), ranging from the coordination of services between two or more services in different locations to the integration of service provider processes (e.g., sharing administrative and financing systems), to fully integrated programs provided in one location (12). Each model requires different resources and operational processes and may have different impacts on individuals’ outcomes. Yet, research on the effective integration of SUHMH services and how to operationalize them to improve individuals’ outcomes remains limited (13).

A recent systematic review of 28 studies on the impact of physically co-locating SUHMH services on the health outcomes of individuals receiving these services indicated that co-located integrated services were consistently correlated with improved patterns of treatment engagement and remission or abstinence from substance use (14). In addition, there was modest evidence indicating that co-located SUHMH services were associated with reduced mental health symptom severity, decreased rates of emergency department presentations and hospitalizations, and improved quality of life. (14). However, most previous studies had a relatively high risk of bias and analysis could not isolate the independent effect of physical co-location from other aspects of integrated care models (e.g., integration of service processes, care coordination) (14). In addition, most previous studies on SUHMH services and support are focused on general populations of individuals receiving these services. There is limited data on outcomes of specific groups of people who access and receive these services, particularly those who are mostly exposed to marginalization, discrimination, and injustices (15).

Individuals who experience the greatest health and social inequities often have more challenges receiving adequate health care services and have poorer experiences in the health care system, particularly due to biases and stigma (16, 17). Findings from previous reviews of scientific and grey literature and a study with people with lived or living experiences (PLLEs) (18–20) indicated that there is limited evidence showing that all individuals receiving SUHMH services meaningfully engage and equally benefit from the current integrated SUHMH services. In particular, the unique needs and experiences of individuals facing significant structural inequities in the health care system, i.e., equity-deserving (ED) populations, have often not been considered in the process of integrating and implementing SUHMH services. ED populations are communities who experience significant collective barriers and multiple layers of oppression. They have historically been denied equal access to employment, education, health care or other opportunities to thrive based on their age, ethnicity, nationality, race, disability, gender identity and expression, sexual orientation, economic status, etc. (21). Other groups who experience significant systemic discrimination and barriers in accessing and receiving proper heath care are Indigenous Peoples in Canada, including First Nations, Inuit and Métis (FNIM). However, since many of FNIM individuals and communities do not identify with or want to be considered ED populations, these three distinct groups are named independently. Many of these different social identities and structures intersect to shape individual and group experiences. Intersectionality is not just about the sum of social identities (e.g., race, gender, socioeconomic status), but about how these identities interact within systems of power and oppression which may impact individual’s experiences and outcomes of various services and support.

To improve access to quality services for these populations, many SUHMH services and support in Canada have sought to increase the use of equity-oriented care in their practices, such as trainings on trauma-informed care and sensitivity to cultural differences. However, it is not clear whether and how these practices influence the outcomes of individuals with multiple intersections receiving SUHMH services. To address these gaps in the literature, the Canadian Centre on Substance Use and Addiction (CCSA) is conducting a pilot study to better understand the specific outcomes of individuals receiving integrated SUHMH services in relation to their intersectionality status. This study focuses on the following five ED groups: 2SLGBTQIA+, newcomers to Canada, visible minorities, persons with disabilities and women. Since many people may identify with more than one FNIM and ED group (e.g., being a newcomer to Canada and woman), the analysis considers the moderating effect of a linear score, ranging from identifying with no FNIM and ED groups to identifying with one or multiple groups (i.e. intersectionality score). The main aim of this pilot study is to evaluate the changes in the clinical and social outcomes of individuals receiving integrated SUHMH services at baseline and three months later in relation to their intersectionality score. We are interested to understand whether the outcomes of individuals who are not in any FNIM and ED groups or who have fewer intersections (i.e., identify with less groups) differ from those with more FNIM and ED intersections (i.e., higher scores).

A second objective of this study also aims to explore whether two qualities of the providing center have an impact on individuals’ outcomes in relation to their intersectionality score: (1) the implementation of key dimensions of equity-oriented care in the services and (2) the level and capability to provide services and support to individuals with cooccurring mental health and substance use disorders (level of integrated SUHMH services). This study recruits staff members who provide services and support, in a variety of roles, to service participants to rate their center on these qualities.

Third, we incorporate the expertise of PLLEs to seek feedback on a) the study results to ensure the findings resonate with individuals receiving SUHMH services and b) the outcome measures used to ensure that they are meaningful and reflective of the experiences of PLLEs in the community. To meet this goal, a smaller group of participants from those who complete the two-time assessment sessions are invited to participate in a post-data analysis validation consultation session with the research team.

## 2 Methods

At the time of writing this manuscript, the baseline data collection has been completed (March 22, 2024). The follow up assessment begins at three months (June 2024).

### 2.1 Study design

This study is a pilot longitudinal study assessing the outcomes of individuals accessing Oasis program at the Sandy Hill Community Health Centre for SUHMH concerns at baseline and at the three-month follow-up. Sandy Hill Community Health Centre is located in Ottawa, Ontario. The Oasis program provides various harm reduction-based health and social services (including HIV and Hep C treatment) for people who use substances and experience barriers to health and recovery due to stigma, poverty, criminalization, and mental illness. The Oasis program provides various SUHMH services in the same location, such as access to supervised consumption services, opioid agonist treatment (OAT), counselling or psychosocial supports, crisis counselling and access to a psychiatrist.

### 2.2 Participant recruitment and eligibility

#### 2.2.1 Service participants

We recruit 100 individuals who utilize SUHMH services and support (i.e., service participants) from the Oasis program. Participants must meet the following inclusion criteria to participate in the study: must be over the age of 18, must receive SUHMH services from the Oasis program, are literate in English (be able to read and write at a Grade 7 level), and be in a stable physical and mental status to participate in the study. Eligible individuals are invited to participate in the two assessment sessions.

From those who complete the two-time assessment sessions and interested in participation in one post-data analysis consultation session, we invite twelve service participants at random. We give selected participants a week to respond before contacting the next interested participants. Twelve service participants who accept and sign the consent form provide their perspectives on the research findings and the outcome measures used.

#### 2.2.2 Staff participants

Five staff members from the Oasis program are invited to participate in the study. Staff participants include those providing services to service participants in a variety of roles, such as nurses, physicians, and peer workers, to reflect their diverse experiences providing services in the center.

### 2.3 Procedure

#### 2.3.1 Consultation with the center providing SUHMH services

Before starting the study, designated people from the providing center (i.e., the Oasis team) reviewed and approved all materials including the protocol, assessment materials (e.g., questionnaires), and consent forms. Certain questions or questionnaires that were identified as inappropriate or trauma-triggering for the specific population visited in this center were removed or replaced before finalizing the assessment package. In collaboration with the Oasis team, we developed a crisis protocol in the event participation in the study causes any distress to the participants.

#### 2.3.2 Engagement plan for people with lived or living experiences

We provide funding to the Oasis program to hire the community workers, PLLEs who work at the center, to support the study’s research activities. They closely collaborate with the research assistant to recruit and signup participants for the study, follow up for the three-month assessment, and support crisis intervention as needed during assessment sessions. This engagement plan is designed to foster a collaborative and inclusive process, ensuring that the study activities are supported by the lived experiences of the community, recognizing the invaluable expertise that PLLEs hold and their pivotal role in establishing trust and rapport with their peers.

#### 2.3.3 Two-time longitudinal assessments of service participants

Service participants are assessed at baseline and again at the three-month follow-up. With help from the Oasis staff, we distribute flyers and invite individuals accessing services and support in the center to participate in the study (N = 100). Interested participants provide written consent for their participation in the study before starting the first assessment session. The recruitment and assessment of one hundred service participants at baseline were started on February 29, 2024, and completed on March 22, 2024. Three months after the baseline assessment (June 2024), participants are informed via their preferred method of access (i.e., email, Phone, or staff of the Oasis program) that they can ask to make an appointment with research team to participate in the second assessment session.

During both the baseline and follow-up assessment sessions, a research assistant readies the participant before starting the assessment. This includes giving instructions, explaining study goals and procedures for participants, and answering any questions. The research assistant reads the questionnaires to the participants and asks them to provide their answer to each question. If participants no longer wish to continue with questionnaires, they are free to leave the room at any time. Staff onsite and a community worker are always there to provide assistance if participants seek additional support or counselling. Before starting the assessment, the research assistant reviews these options with participants. Each assessment session takes approximately one hour. Participants are compensated CAD50 at each of the two assessment points (total CAD100). This information is provided in the informed consent form.

#### 2.3.4 Assessment of staff participants

Five staff members from the providing center are invited to participate in the study and rate their program’s state of integrating equity-oriented care and capability to provide integrated SUHMH services. We ask the Oasis program manager to send out an email to the staff members and ask interested individuals to connect with study’s research assistant to participate in the study. We recruited one staff member in each of the following job groups to complete the questionnaires: nurses, physicians, mental health clinicians, harm reduction workers and, peer workers. Responses from staff participants remain confidential and an average of their responses will be used in the analysis to protect confidentiality and reduce the risk of bias in responding. The assessments take approximately one hour to complete. The staff participants complete the questionnaires on their own (time) at baseline and are compensated CAD50 for their participation. The recruitment and assessment of five staff participants were started and completed during the month of March 2024.

#### 2.3.5 Post-data analysis consultation

Since this is a pilot study, the team seeks feedback on both the study results and the outcome measures used to apply lessons learned for the scale up of the study in other contexts. This phase involves offering validation sessions where a group of twelve service participants can provide insights and perspectives on the research findings and other analysis/variables that should be considered. This approach is used to validate whether the conclusions are appropriate and/or provide alternative interpretations and implications. The participants will be purposely selected to match the proportion of FNIM and ED groups from the two-time assessment to ensure representation. Examples of questions asked will include: *Does your experience match up with what was found in the study?; What parts of the findings resonated the most?; Do you think there is anything about your experiences that the findings didn’t catch?; What factors (e.g., cultural, social, financial) do affect your experiences when accessing SUHMH services?; How do factors such as your race, gender, or disability intersect to shape your access and experiences of SUHMH services?* This approach ensures that the study accurately reflects the experiences of participants. The collaborative approach of this engagement plan underscores the commitment to inclusivity and relevance in the pursuit of improved healthcare outcomes for ED groups.

#### 2.3.6 Duration of the research project

The pilot project, including recruitment, two-time assessments, data analysis, writing of reports, and knowledge mobilization activities, lasts for 12 months starting in February 2024.

### 2.4 Outcome measures

#### 2.4.1 Assessment of service participants

##### 2.4.1.1 Equity deserving questionnaire

To identify the FNIM and ED status of participants, the most updated version of the self-identification questionnaire developed by the three Canadian federal research funding agencies is used. The questionnaire covers eight dimensions: age, gender identity, sexual orientation, FNIM identity, racialized identity, population group, disability, and language. The research team added two questions to identify newcomer status based on the definition introduced by Statistics Canada in 2006. Recent immigrants (also known as newcomers) refer to landed immigrants who came to Canada up to five years prior to a given census year (Statistics Canada, 2006).

##### 2.4.1.2 International consortium for health outcomes measurement

The ICHOM Addiction set is used to assess several outcome measures related to substance use and addictive behaviors, including demographic factors, frequency and quantity of substance use, severity of dependence, symptoms of post-traumatic stress disorder (PTSD), global functioning, and quality of life. The ICHOM Patient-Centered Outcome Measures for Addiction (22) is developed based on a globally applicable, minimum standard set of outcome measures for people who seek treatment for substance use and mental illnesses. This outcome measure set is the result of collaboration among leading physicians, measurement experts, and people with lived experiences.

##### 2.4.1.3 Modified mini screen

MMS (23) is used to screen for mood, anxiety, and psychotic spectrum disorders. The original questionnaire has twenty-two questions with yes/no responses. We removed two questions related to the experience of trauma because of the overlap with questions included in the ICHOM.

##### 2.4.1.4 Current and past use of substance use and mental health services and support

Two separate tables are developed specifically for this study in collaboration with the providing center (i.e., Oasis team). These tables assess participants’ use of substance use and mental health services and support in the past year (at baseline assessment) and past 90 days (at follow-up assessment) including services received in the current center and elsewhere. This includes the type of services, such as harm reduction services for substance use health and psychotherapy for mental health, and the number of days in each service.

##### 2.4.1.5 Assessment of needs

A short questionnaire including open-ended questions is developed specifically for this study in collaboration with the providing center (i.e., Oasis team). This is used to assess participants’ needs to receive various services, such as emotional and mental health, physical health, and housing.

#### 2.4.2 Assessment of staff members

##### 2.4.2.1 EQUIP rate your organization tools

Four tools are used to assess providing center’s actions and capacity for equity-oriented care. The first one, which includes ten items, assesses organizations in terms of their general capacity and integration level of equity-oriented health services (24). The other three, each including ten items, are designed to assess organizations on three key dimensions of equity-oriented health care: addressing anti-Indigenous racism (25), harm reduction and reducing substance use stigma (26), and trauma- and violence-informed care (27). These tools have generally been used to enhance the capacity to provide equity-oriented services in different health care settings (e.g., 28, 29).

##### 2.4.2.2 Dual diagnosis capability in addiction treatment version 4.0

DDCAT (30) measures the ability of substance use providers to meet the needs of individuals with cooccurring mental health and substance use disorders. The DDCAT evaluates 35 program elements that are subdivided into seven dimensions: program structure, program milieu, clinical process (assessment and treatment), continuity of care, staffing, and training. Previous studies have shown support for the psychometric properties of the measures (e.g., inter-rater reliability, internal consistency, and sensitivity to change) (30).

## 3 Data management and analysis

The participants’ responses to the questionnaires and interviews (both service participants and staff participants) will be entered into a Statistical Package for the Social Sciences (SPSS) by the study’s research assistant. The data will be cleaned, and quality checked before data analysis. Descriptive statistics will be utilized to describe the sample at baseline and at the three-month follow-up.

A series of multilevel generalized estimating equations (GEEs) will be conducted to examine the change in each outcome measure (e.g., change in substance use rate, mental health symptoms, quality of life). GEE accounts for the correlation among repeated measures and examines the relationships between different variables within the model at multiple time points simultaneously. Outcome analyses will test whether the reported outcome rates change over the 3-month period, when a linear score for intersectionality score (ranging from identifying with no FNIM and ED groups to identifying with one or multiple groups) is entered as the moderator. GEE analyses will control for potential confounding variables (e.g., length of stay in current services, number and type of services received inside and outside of the providing center).

In the following steps, separate analyses will be utilized in which the center’s score on EQUIP (level of integration of equity-oriented care) and DDCAT (center’s capability to meet the needs of individuals with cooccurring MH disorders) will be entered in the analyses as moderators to test whether the change in reported outcomes of individuals differ based on the center’s qualities on these scores.

Qualitative thematic analysis will be used for summarizing and interpreting the findings of the post-data analysis consultation.

## 4 Ethics and Dissemination

### 4.1 Research ethics approval

This pilot study is approved by the Advarra Institutional Review Board (IRB) (Pro00076474).

### 4.2 Consent and confidentiality

Ensuring the safety and well-being of participants is a top priority. Prior to participation, all potential participants receive a detailed explanation of the study’s purpose, procedures, potential risks, and benefits. They are given multiple opportunities to ask questions and provide informed consent voluntarily. The consent forms clearly outline the use of their data and their right to withdraw from the study at any time without repercussions. Service participants are reassured that their decision to participate in the study or to withdraw from the study at any time do not have any negative consequences to the medical care or other services to which they are entitled or are presently receiving.

Both service and staff participants are asked to provide written informed consent before participating in the study. This includes signing (1) one informed consent form for participation in two-time assessment sessions for service participants, (2) one informed consent form for participation in the post-data analysis consultation session for a sub-group of service participants, and (3) one informed consent form for participation in one assessment session for staff participants.

The names of the participants are not used in any discussion or correspondence about the data. The findings of this study do not appear in participants’ records or files. No information that discloses participants’ identities are released or published and no records that identify participants by name or initials are allowed to leave the research investigators’ offices.

### 4.3 Access to data

All data are confidential, where participants’ data are only stored using unique research IDs securely in electronic and/or physical formats. Electronic data are stored on password-protected and encrypted devices, while physical records are kept in locked storage at the research team’s office, with access only by the study team. Electronic files are kept for at least five years and possibly longer. Paper (physical) materials are stored for five years following the completion of the study in locked cabinets at the research team’s office where access is limited, after which point these materials will be destroyed securely. Data sharing is restricted. De-identified electronic files containing quantitative and qualitative data are only used for data analysis.

### 4.4 Dissemination and knowledge mobilization plans

The study team will seek to disseminate findings through publishing in academic journals, presenting at conferences, developing policy briefs, and sharing recommendations and next steps with SUHMH services providers across Canada. The study team will also create a knowledge mobilization plan that will engage with appropriate partners and different levels (e.g., front line service providers, jurisdictional and federal decision makers) to ensure that tailored and key messaging is not only received but also implemented by the appropriate audience. Various knowledge products may be created as a result.

## 5 Discussion

FNIM and ED individuals with substance use disorders and mental illnesses may have different experiences regarding access, treatment engagement, clinical and social outcomes, and quality of life before, during and after receiving SUHMH services and support. The current study seeks to better understand the outcomes of individuals receiving integrated SUHMH services in relation to their intersectionality status. The results of this study will particularly show whether an individual’s intersectionality score, ranging from identifying with no FNIM and ED groups to identifying with one or multiple groups, impact their outcomes over a course of three months receiving such services. The findings and lessons learned may be used to improve practices and inform policies to better address the specific needs of different FNIM and ED groups. The findings of this study will inform integrated SUHMH community services by emphasizing equity and inclusive approaches.

This pilot study has several limitations. First, this research is being conducted in a single community health center providing specific SUHMH services and support. Therefore, the findings from this study may not be generalizable to other populations or other SUHMH settings providing services and support. However, the findings of this study will be used to inform other contexts and regions for future phases of the project, including future research with settings of varying degrees and models of service integration. Second, we expect that the rate of attrition will be high in the second follow-up assessment which may affect the evaluation of changes in service participants’ outcomes. This was mitigated by gathering as large a sample size as possible at baseline, to allow sufficient power for follow up analyses even if significant attrition occurs. Third, the small number of participants in this study will not allow for disaggregation of the data to compare each FNIM and ED group with others or explore within each FNIM and ED group (e.g., transgender people in 2SLGBTQIA+ group). Finally, for this study, we focus on ED groups that are traditionally known to experience high levels of collective barriers and discrimination in the health care system and society in general. However, there may be other groups outside of these communities that are also affected by similar inequity in accessing and receiving health care services. Future research should identify and include other less known and studied groups to ensure greater inclusivity.

## Data Availability

The raw data supporting the conclusions of this article will be made available by the authors, without undue reservation.
